# Genetic Diversity of *Listeria monocytogenes* Isolated From Three Commercial Tree Fruit Packinghouses and Evidence of Persistent and Transient Contamination

**DOI:** 10.3389/fmicb.2021.756688

**Published:** 2022-01-10

**Authors:** Yi Chen, Tobin Simonetti, Kari Peter, Qing Jin, Eric Brown, Luke F. LaBorde, Dumitru Macarisin

**Affiliations:** ^1^Center for Food Safety and Applied Nutrition, Food and Drug Administration (FDA), College Park, MD, United States; ^2^Department of Food Science, Pennsylvania State University, University Park, PA, United States; ^3^Fruit Research and Extension Center, Pennsylvania State University, University Park, PA, United States

**Keywords:** Genomics, diversity, *Listeria*, packinghouse, apple

## Abstract

Whole genome analysis was performed on 501 isolates obtained from a previous survey which recovered 139 positive environmental sponge samples (i.e., up to 4 isolates per sample) from a total of 719 samples collected at 40 standardized sites in 3 commercial apple packinghouse facilities (i.e., P1, P2, and P3) over 3 successive seasons in a single production year. After excluding duplicated isolates, the data from 156 isolates revealed the clonal diversity of *L. monocytogenes* and allowed the detection of transient contamination, persistent contamination, and cross-area transmission events. Facility P2 with the poorest sanitary conditions had the least diversity (Shannon’s index of 0.38). P2 contained a Clonal Complex (CC) 554, serogroup IVb-v1 strain that persisted throughout the year and spread across the entire facility, a singleton Sequence Type (ST) 1003, lineage III strain that persisted through two seasons and spread across two areas of the facility, and 3 other clones from transient contaminations. P1 and P3, facilities with better sanitary conditions, had much higher diversity (i.e., 15 clones with a Shannon’s index of 2.49 and 10 clones with a Shannon’s index of 2.10, respectively) that were the result of transient contamination. Facilities P1 and P3 had the highest incidence (43.1%) of lineage III isolates, followed by lineage I (31.3%) and lineage II (25.5%) isolates. Only 1 isolate in the three facilities contained a premature stop codon in virulence gene *inlA*. Fourteen samples yielded 2–3 clones per sample, demonstrating the importance of choosing appropriate methodologies and selecting a sufficient number of isolates per sample for studying *L. monocytogenes* diversity. Only 1 isolate, belonging to CC5 and from facility P3, contained a known plasmid, and this was also the only isolate containing benzalkonium chloride tolerance genes. The persistent CC554 strain did not exhibit stronger sanitizer resistance than other isolates and did not contain any confirmed molecular determinants of *L. monocytogenes* stress resistance that were differentially present in other isolates, such as genes involved in sanitizer tolerance, heavy metal resistance, biofilm-forming, stress survival islet 1 (SSI-1), stress survival islet 2 (SSI-2) or *Listeria* genomic island (LGI2).

## Introduction

*Listeria monocytogenes* is estimated to cause over 1,600 illnesses and 250 deaths annually in the United States, making it one of the leading causes of death from foodborne illness (Silk et al., 2012). Older adults, persons with immunocompromising conditions, and pregnant women and their newborns are at higher risk of listeriosis than the general population (Silk et al., 2012). Most listeriosis outbreaks have historically been linked to ready-to-eat meats and dairy products (Silk et al., 2012). However, produce-associated listeriosis outbreaks are increasingly recognized (Chen et al., 2017c).

Traceback investigations in a caramel apple outbreak in 2014–2015 converged on a single apple grower, and samples from the apple packing facility and apples in the distribution chain yielded *L. monocytogenes* isolates highly related to patient isolates by whole genome sequencing (Angelo et al., 2017). The relatively large size of this outbreak (35 cases in 7 states) suggests persistence or repeated contamination of *L. monocytogenes* that allowed contamination of whole apples used to make caramel apples. Although fresh produce is now a well-established source of listeriosis, apples were an unexpected vehicle, given their high acidity that generally does not support *L. monocytogenes* growth. However, *L. monocytogenes* have been shown to survive for extended periods of time on experimentally contaminated Red Delicious, Fuji and Granny Smith apples and that the common practice of waxing could facilitate long-term survival of *L. monocytogenes* on apples (Macarisin et al., 2019).

Food safety research on tree fruits is not as extensive as that on leafy greens and vegetables, because these fruits are generally not in direct contact with soil, irrigation water, or organic fertilizers. The risks of apple contamination associated with current pre- and post-harvest practices, as well as environmental niches for *L. monocytogenes* in tree fruit production continuum, represents a large knowledge gap. A better understanding of the ecology, distribution, and persistence of *L. monocytogenes* in the tree fruit production environments is paramount to gaining a greater understanding of the sources and mechanisms of contamination.

We earlier had hypothesized that fruit packing/storage facilities can harbor transient and/or persistent *L. monocytogenes* strains leading to post-harvest contamination of fruits. We therefore conducted a 2-year environmental survey to obtain data on the incidence and prevalence of *L. monocytogenes* in 3 commercial apple packing and storage facilities (Simonetti et al., 2021). Samples were collected from fruit washing, waxing, sorting equipment and surroundings, cold storage rooms, walls, and floors. *L. monocytogenes* was most often found in the packing line areas, where moisture and fruit debris were commonly observed and less often in dry cold storage and packaging areas. Transient contamination was referred to contaminations that were detected in only one or two samplings and later eliminated. Persistent contamination was referred to those that occurred throughout entire sampling period. Persistent contamination was attributed to poor sanitation practices, including the inability of water drainage systems to prevent moisture accumulation on floors and equipment during peak production times and uncontrolled employee and equipment traffic throughout the facility (Simonetti et al., 2021). For the first year of that study, 139 positive samples from a total of 719 sponge samples were recovered yielding 501 isolates (i.e., up to 4 isolates per positive sponge sample). In the present study, our objective was to perform whole genome sequencing of these isolates to obtain highly resolved geo-spatial source distribution and genetic relationships among isolates.

## Materials and Methods

### Isolates

Isolates analyzed in this study were obtained previously from 40 standardized non-food-contact surface locations in 3 packinghouses at two different times each within the fall depending on the facility (F1, September and F2, October or November), winter (W1, January or February and W2, February or March), and spring (S1, May and S2, June or July) seasons in the 2016–2017 production year (Table 1; Simonetti et al., 2021). Detailed descriptions of the conditions of each facility, sampling sites and dates, and *L. monocytogenes* detection and isolation methods were described in detail by Simonetti et al. (2021). Briefly, samples were enriched in Buffered *Listeria* Enrichment Broth (BLEB) (Oxoid Ltd., Basingstoke, Hamsphire, United Kingdom) for 4 h at 30°C. After the initial incubation, *Listeria* Selective Enrichment Supplement (SR0149A, Thermo Fisher Scientific, Lenexa, KS) was added to each sample and incubated for an additional 44 h at 30°C. The enriched samples were streaked (10 μl) onto Agar *Listeria* Ottaviani and Agosti (ALOA) and RAPID’ *L. monocytogenes* (RLM) (BioRad, Hercules, CA) media, then incubated for 24–48 h at 37°C. Presumptive *L. monocytogenes* isolates were confirmed with Qiagen Multiplex PCR kits (Qiagen Inc., Germantown, MD) using *iap* and *lmo2234* primers specific for *Listeria* spp. and *L. monocytogenes*, respectively, as described by Chen and Knabel (2007).

**TABLE 1:** Data from Simonetti et al. (2021).

Area	Sub-area	Sample site description	Zone	Location ID#
Cold storage	Short-term and staging^a^	Floor 1/3^b^	3	01
		Floor 2/3	3	02
		Floor 3/3	3	03
		Floor crack/seam 1/2	3	04
		Floor crack/seam 2/2	3	05
		Foot of fruit storage bin	3	06
	Long-term	Floor 1/3	3	07
		Floor 2/3	3	08
		Floor 3/3	3	09
		Floor crack/seam	3	10
		Foot of fruit storage bin	3	11
Packing line	Dump tank	Rim above water line 1/2	2	12
		Rim above water line 2/2	2	13
		Bin loading equipment	2	14
		Bin unloading equipment	2	15
		Interior of cull bin	3	16
	Spray-wash	Spray or structural bar over brushes	2	17
		Drip pan	3	18
		Scraper or structural bar under brushes	2	19
		Floor directly below line	3	20
	Fan-dry	Structural support/flow partitions above the line	2	21
		Drip pan	3	22
		Scraper bars and drip pan funnel below line	3	23
		Floor directly below the line	3	24
	Wax	Structural support/wax spray bar over brushes	2	25
		Flow partition dividers/wax drip area	2	26
		Drip pan/wax drip area	3	27
		Structural support/wax drip area below the line	3	28
		Floor directly below line	3	29
	Potential cross-contamination sites	Catwalks adjacent to packing line 1/2	3	30
		Catwalks adjacent to packing line 2/2	3	31
		Adjacent floor drains along the packing line	3	32
		High traffic floor adjacent to packing line 1/2	3	33
		High traffic floor adjacent to packing line 2/2	3	34
		Forklift tine	3	35
		Forklift wheel	3	36
Packaging line		Floor directly below sorting line	3	37
		Sorting line equipment	2	38
		Floor directly below packaging line	3	39
		Line equipment	2	40

*Facility areas, sub-areas, and forty standardized sites sampled in each of 3 packinghouses. A total of six sampling events were conducted: September and November 2016 (fall sampling), January and March 2017 (winter sampling), and May and June 2017 (spring sampling).*

*^a^Short-term storage sub-areas refer to staging areas around the packing line where apples coming in from the orchards become temporarily backed up. The apples in this area could go directly to the packing line or get moved to the long-term storage sub-areas, which are large, refrigerated rooms on site where the apples would eventually be moved back to the packing line.*

*^b^1/3 indicates one of the three samples that were taken from three different locations of each sample site in each sub-area.*

### Whole Genome Sequencing

All isolates were sequenced on an Illumina MiSeq platform (250-bp, paired-end reads) (Illumina, Inc., San Diego, CA) using the Nextera XT Library Preparation Kit per the manufacturer’s instructions. The genomic sequence contigs for each isolate were *de novo* assembled using Qiagen CLC Genomics Workbench 11.1 (Aarhus, Denmark). We then analyzed these genomes by a previously developed cgMLST typing scheme implemented in Ridom SeqSphere + (Ridom^©^ GmbH, Germany), targeting 1,827 core genes of *L. monocytogenes* (Chen et al., 2016b). A neighbor-joining tree was constructed using the pairwise allele differences. The combination of clustering in the neighbor-joining tree and number of allele differences was used to identify transmission and persistence events.

### Determination of Clonal Group, Serogroup, and Lineage

*In silico* MLST implemented in the SeqSphere + software was used to determine the sequence type of these isolates. Clonal complex (CC) and singleton were then assigned based on the definition by Ragon et al. (2008) and profiles curated in the Institute Pasteur MLST *Listeria* database.^1^ A CC is defined by the 7-gene multilocus sequence tying (MLST) scheme (Ragon et al., 2008) as a group of sequence types (STs) differing by no more than one allele from at least one other ST in the group, and a group having only one ST which differs from all other existing STs in the database by at least two alleles is defined as a singleton (Ragon et al., 2008). *In silico* serogroup identification was performed by determining the presence of genetic markers for the four major serogroups: IIa (1/2a or 3a); IIc (1/2c or 3c); IVb (4b, 4d or 4e), and IIb (1/2b or 3b) (Doumith et al., 2004). Although designation of those molecular serogroups (e.g., 1/2a, 3a), instead of serotypes (e.g., 1/2a) were achievable based on the WGS data, one serotype in each serogroup (e.g., 1/2a, 4b, 1/2b) is typically more prevalent. Lineage I or lineage II isolates were determined using the combination of serogroup information and cgMLST phylogeny. Lineage III and/or IV isolates were determined using cgMLST phylogeny.

### Determination of the Presence of Select Virulence Genes and Genes Implicated in Stress Resistance, Premature Stop Codon in *inlA* and Plasmid

BLAST was performed to determine the presence of select virulence and persistence genes among all the isolates. A threshold of ≥ 70% query coverage with ≥ 80% sequence identity of BLAST alignment indicated the presence of a gene or genomic island. The genes analyzed in this study included those in major *Listeria* pathogenicity islands and stress survival islets, those encoding internalins, including inlA, and those associated with sanitizer (i.e., quaternary ammonium compounds [QAC]) and heavy metal resistance, and tolerance to various adverse conditions (Moura et al., 2016; Maury et al., 2019). The *inlA* sequences were extracted from the whole genome sequences using CLC Genomics Workbench and aligned using MEGA 7.0; and premature stop codons (PMSCs) were determined manually. The contigs of each shotgun genome were compared with complete sequences of 60 *Listeria* plasmids deposited in the GenBank as of November 10, 2019 by BLASTn (Camacho et al., 2009) and the plasmid *repA* gene was compared with all shotgun genomes by BLASTn (Camacho et al., 2009). We considered a contig as a plasmid contig if it contained ≥ 60% of any plasmid sequences. We considered *repA* present in a shotgun genome if the BLAST had query coverage of ≥ 60% and sequence identity of ≥ 60%. Because 90% of the complete plasmids are ≥ 10 Kbp and the shortest plasmid length is 3.7 kb, we determined that a shotgun genome contained a plasmid if the total length of plasmid contigs in a shotgun genome exceeded 10 Kbp and *repA* was present in a plasmid contig. If the total length of plasmid contigs in a shotgun genome was less than 10 Kbp but exceeded 3.7 Kbp, we considered the result inconclusive.

### Statistical Analysis of Genotype Distributions

For statistical analysis, we removed duplicate isolates from the same sample. During each sampling, 40 samples were taken and up to 4 typical colonies were picked from each sample for identification and WGS. If the 4 isolates from the same sample likely belonged to the same strain, we removed the duplication of 3 isolates from further analysis. If isolates from different samples likely belonged to the same strain, they were not considered duplicates. We calculated the percentage of isolates belonging to each lineage, serotype, and clones. We also calculated the Shannon’s index among isolates from different facilities using previously described methods; Shannon’s index measures the diversity of a strain collection (Shannon, 1948).

### Growth in the Presence of Low Concentrations of Sanitizer

The resistance of a set of representative strains to low levels of Sani-T-10, a QAC-based sanitizer used by facility P2, was compared using a method adapted from Harrand et al. (2020), which suggested that the currently recognized QAC resistance genes of *L. monocytogenes* only confer resistance to low levels of QAC, not high levels. Sani-T-10 contains 5% of n-alkyl (60% C14, 30% C16, 5% C12, 5% C18) dimethyl benzyl ammonium chloride and 5% of n-alkyl (68% C12, 32% C14) dimethyl ethylbenzyl ammonium chloride. Final concentrations used for growth experiments included 1, 2, 3, 4, 5, 6, and 7 ppm. Sani-T-10 was prepared in BHI broth, and 100-μl overnight culture in BHI were then added to 7 ml BHI broth containing Sani-T-10 before incubation at 30°C for 24 h. The minimum inhibitory concentration (MIC) was defined as the lowest sanitizer concentration at which *L. monocytogenes* growth was inhibited. The growth was measured by OD_600_ with 0.010 as the threshold.

## Results and Discussion

We performed whole genome sequence analysis of a total of 501 isolates obtained from the survey. After determining the genetic relatedness of all the isolate and removing duplicate isolates (i.e., isolates from the same swab sample that likely belonged to the same strain), we analyzed 156 isolates in detail. The NCBI Biosample ID, ST, CC, genetic lineage, molecular serogroup, facility ID, sampling date, location number and area designation listed in Supplementary Table 1 for the 156 isolates and in Supplementary Table 2 for the 501 isolates. Even though isolates belonging to epidemiologically-defined outbreaks could have varying degrees of genetic diversity (Chen et al., 2016b), our determination of duplicate isolates were not affected by the threshold of WGS diversity used to define a strain because when two or more isolates of the same sample in the current study belonged to the same clone, they always had ≤ 4 allele differences and thus were considered duplicates. When two or more isolates of the same sample differed by more than 4 alleles, they always belonged to different clones and thus were obviously different strains. In 125 of the 139 positive samples, all isolates of each sample differed by ≤ 4 alleles; in 11 positive samples, each sample yielded 2 strains; and in 3 positive samples, each sample yielded 3 strains. Overall, 345 isolates were removed as duplicates and the remaining 156 isolates were subjected to further analyses.

**FIGURE 1:**
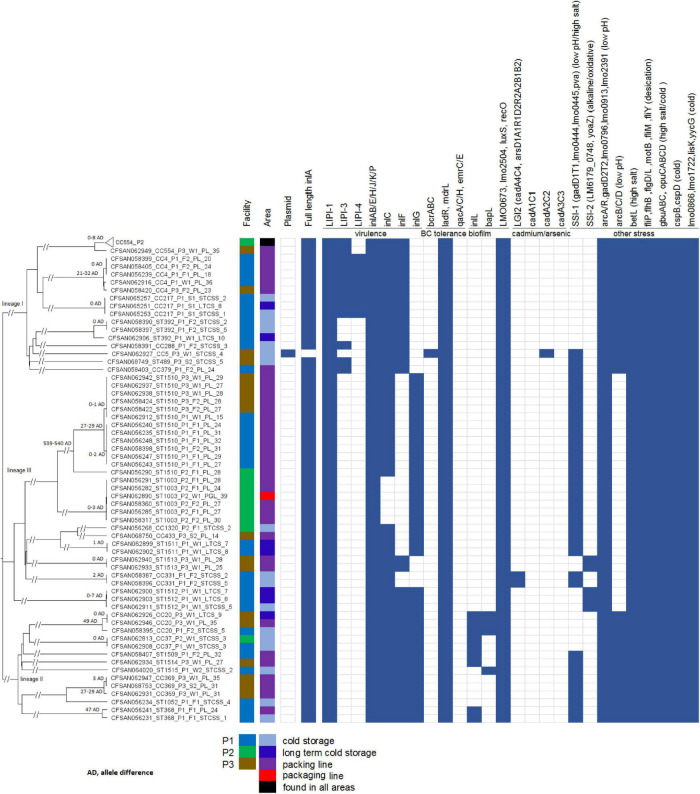
Neighbor-joining tree of the 156 deduplicated isolates collected in 3 apple packinghouses. The Biosample ID is followed by clonal complex or singleton designation, facility number, sampling, sub-area of the facility (i.e., STCSS, short-term cold storage and staging; LTCS, long-term cold storage; PL, packing line; PGL, packaging line) and the number corresponding to each subarea of the facility. The 96 CC554 isolates in facility P2 are collapsed into a triangle shown as the top taxa of the tree. The presence (solid blue color) and absence (empty box) of genes associated with virulence and stress resistance are shown. The pairwise allele differences (AD) of isolates in each clade are shown near the root of the clade.

We determined the genetic relatedness among isolates from different samples to identify possible persistence and transmission events and this determination was not significantly affected by the threshold of WGS diversity. When isolates from different samples belonged to the same clone, the following scenarios were observed (Figure 1). In 12 clades, isolates differed by ≤ 3 alleles; in 1 clade, 3 ST1512 isolates differed by 0, 7, and 7 alleles in a pairwise comparison; we determined each clade to likely represent one strain. The 96 CC554 isolates in one clade differed by up to 8 alleles, and any neighboring isolates in a minimum-spanning tree (tree not shown) differed by 0–4 alleles. Considering that in previous studies isolates that were likely the same strain usually differed by less than 7 alleles (Chen et al., 2016b; Moura et al., 2016), we determined that this clade of 96 isolates likely represented one strain. All clades with diversity larger than the above-described clades contained isolates that differed by at least 21 alleles in pairwise comparisons. Specifically, the clade of 3 CC4 isolates differed from two other CC4 isolates by 21, 23, and 32 alleles in pairwise comparisons; the 2 ST1510 clades differed by ≥ 27 alleles; the clade of 2 CC369 isolates, exhibiting 2 allele differences, differed from another CC369 isolate by 29 alleles; the 2 ST368 isolates differed by 47 alleles; and the clade of 2 CC20 isolates, exhibiting 0 allele difference, differed by 49 alleles from another CC20 isolate. The isolates in these more diverse clades were relatively close genetically, however, we could not conclude whether they were the same strain and therefore did not use those isolates to infer transmission or persistence. All other more diverse clades contained isolates differing by ≥ 100 alleles, and we considered those isolates to represent different strains.

In the description below, sequence type (ST) is followed by clonal designation [i.e., clonal complex (CC) or singleton] and molecular serogroup (e.g., IIa, IIb, IVb, IVb-v1). If an isolate belonged to a singleton, only ST is listed; if an isolate belonged to a CC, both ST and CC are listed. Serogroup IIb and IVb isolates belong to lineage I; serogroup IIa and IIc isolates belong to lineage II; and lineage III isolates could not be serotyped by molecular methods and were designated as LIII. During the sampling, samples were labeled as #1 through #40.

The sampling locations in each facility were standardized to allow easy comparison and description (Table 1). The details of these standardized sites were previously described (Simonetti et al., 2021). Each facility was divided to 3 areas, cold storage, packing line and packaging line. The packing line is the main processing area that includes washing, sorting, brushing, coating, drying, and grading. The packaging line is where the fruits are packaged into boxes or bags before storage (Simonetti et al., 2021). Each area was further divided to multiple sub-areas and multiple sampling sites were chosen in each sub-area; in some sampling sites, different locations were sampled, which yielded different samples (Table 1). Only deduplicated isolates are discussed below.

### Facility P1 (33 Isolates: 7 Serogroup IIa, 5 IIb, 7 IVb, 7 IVb-v1, and 17 LIII)

#### Two Samplings in Fall 2016, September (F1) and November (F2)

(1) In the F1 sampling, 8 positive samples yielded 9 isolates.
  Two samples in the short-term storage and staging sub-area were positive. Sample #1 from the floor 1/3 near apple input yielded 1 isolate (ST368, IIa) and sample #4 from the floor crack/seam 1/2 yielded 1 isolate (ST1052, IIa). In this article floor 1/3 indicates the first of the 3 samples taken from 3 different locations of the floor sample site (Table 1); floor crack/seam 1/2 indicates that the first of the 2 samples taken from 2 different locations of the floor crack/seam sample site (Table 1).
  Six samples from the packing line were positive. Sample #27 from a waxing drip pan, sample #29 from the floor below waxing, sample #31 from the catwalks adjacent to the packing line 2/2 (a potential cross-contamination site), and sample #32 from adjacent floor drains along the packing line (a potential cross-contamination site) yielded 4 isolates of ST1510 (LIII); sample #24 from the floor below fan-dry yielded 2 isolates (ST1510, LIII and ST368, IIa); and sample #18 from a spray-wash drip pan yielded 1 isolate (ST219/CC4, IVb). Among these isolates, all 5 ST1510 isolates likely belonged to the same strain, indicating cross-contamination between the waxing and fan-dry sub-areas, possibly due to the same strain present in the catwalks going through different sub-areas of the packing line. The drain common to the spray-wash, fan-dry and waxing sub-areas had a convergence of rinse water and product debris, which might explain that the drain was contaminated with the same strain as those packing line sub-areas.
(2) In the F2 sampling, 7 positive samples yielded 11 isolates.
  Three samples in the short-term storage and staging sub-area were positive. Sample #2 from the floor 2/3 at the exit to packing yielded 2 isolates (ST331/CC331, LIII and ST392, IIb); sample #3 from the floor 3/3 at the room center yielded 1 isolate (ST323/CC288, IIb); and sample #5 from floor crack/seam 2/2 yielded 3 isolates (ST331/CC331, LIII; ST392, IIb and ST1508/CC20, IIa). Among these isolates, the CC331 isolates from samples #2 and #5 were likely the same strain; the ST392 isolates from samples #2 and #5 were also likely the same strain. The sample sites in the cold storage area were floors, floor cracks/seams and the feet of fruit storage bins, and therefore, cross-contamination due to foot or equipment traffic was highly possible.
  Four samples in the packing line were positive. Sample #31 from the catwalks adjacent to the packing line 2/2 (a potential cross-contamination site) yielded 1 isolate (ST1510, LIII); sample #20 from the floor below the spray-wash sub-area yielded 1 isolate (ST219/CC4, IVb); sample #24 from the floor below the fan-dry sub-area yielded 2 isolates (ST379/CC379, IIb and ST219/CC4, IVb); and sample #32 from adjacent floor drains along the packing line yielded 1 isolate (ST1509, IIa). Among these isolates, the ST219/CC4 isolates from samples #20 and #24 were likely the same strain.
(3) Combining data from the two fall samplings, the ST1510 isolate from sample #31 from the F1 sampling likely belonged to the same strain as the ST1510 isolates from samples #24, #27, #29, #31 and #32 from the F2 sampling. This indicated that this strain spread across multiple sub-areas of the facility P1 packing line and persisted through F1 and F2 sampling. However, it is also possible that the same strain was reintroduced to the facility from external sources.

#### Two Samplings in Winter 2017, February (W1) and March (W2)

(1) In the W1 sampling, 7 positive samples yielded 9 isolates.
  Five samples in the cold storage area were positive. Sample #10 from a floor crack/seam in long-term storage yielded 1 isolate (ST392, IIb); sample #7 from the floor 1/3 in the long-term storage sub-area yielded 2 isolates (ST1511, LIII and ST1512, LIII); sample #8 from the floor 2/3 in the long-term storage yielded 2 isolates (ST1511, LIII and ST1512, LIII); sample #3 from the floor 3/3 in the short-term staging sub-area yielded 1 isolate (ST37/CC37, IIa); and sample #5 from the floor crack/seam 2/2 in the short-term storage and staging sub-area yielded 1 isolate (ST1512, LIII). Among these isolates, the 2 ST1511 isolates from samples #7 and #8 were likely the same strain, and the 2 ST1522 isolates from samples #7 and #8 were likely the same strain. Among these isolates, the ST1512 isolates from samples #5, #7, and #8 likely belonged to the same strain.
  Two samples in the packing line were positive. Sample #15 from a bin unloading equipment by dump tank yielded 1 isolate (ST1510, LIII). Sample #36 from a forklift wheel (a potential cross-contamination site) yielded 1 isolate (ST219/CC4, IVb).
(2) In the W2 sampling, only sample #2, from the floor 2/3 near exit of apple output in the short-term storage staging sub-area, was positive and yielded 1 isolate (ST1515, IIa).
(3) Combining data from the two samplings, no persistence or reintroduction events were observed.

#### Two Samplings in Spring 2017, May (S1) and June (S2)

(1) In the S1 sampling, 3 samples, from the cold storage area, were positive and yielded 3 isolates that likely belonged to the same strain (ST217/CC217, IVb). These samples were sample #1 from the floor 1/3 in the short-term storage and staging sub-area, sample #2 from the floor 2/3 in the short-term storage and staging and sample #8 from the floor 2/3 in long-term storage sub-area.
(2) In the S2 sampling, no sample yielded *L. monocytogenes*.

#### Summary of P1

**FIGURE 2:**
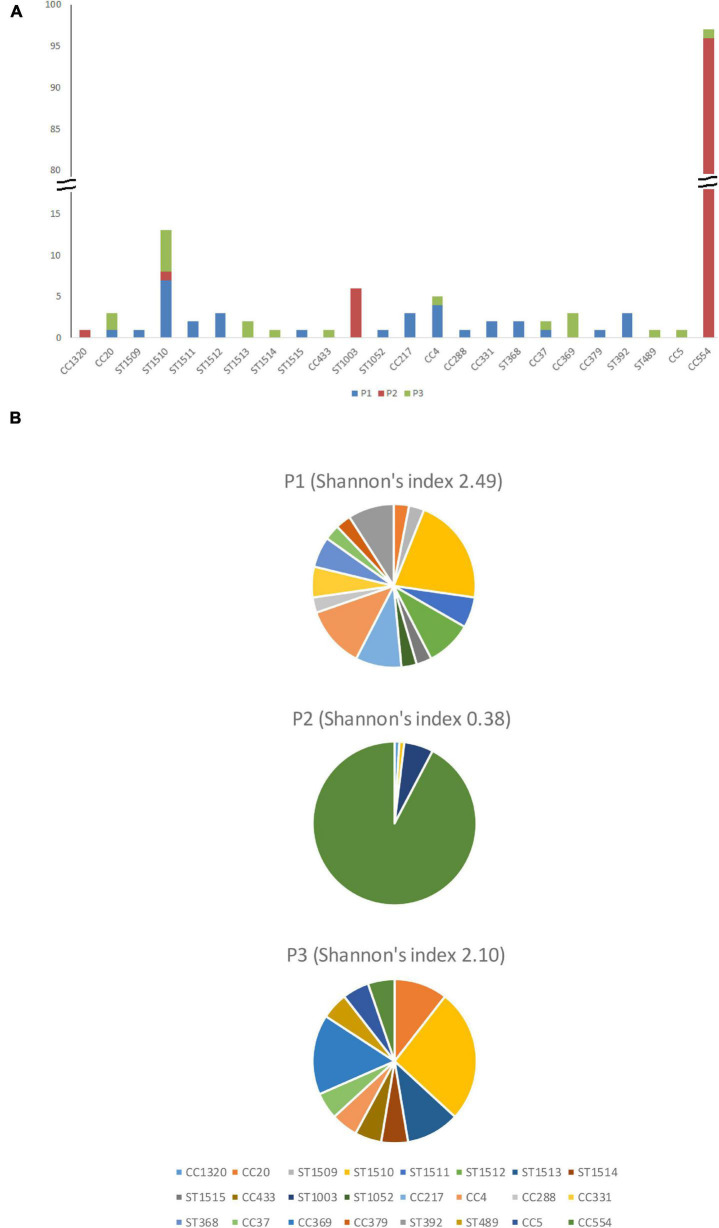
The number **(A)** and percentage **(B)** of isolates from different clonal complexes and singletons in facility P1, P2, and P3.

Overall, 33 isolates from 15 clones were recovered from P1, resulting in a Shannon’s diversity index of 2.49 (Figure 2). No isolate from different seasons belonged to the same strain, indicating no persistence across seasons. Therefore, the contamination was transient. Isolates from the cold storage area and those from the packing line did not belong to the same strain, indicating that the environmental isolates of *L. monocytogenes* did not spread between these two areas, suggesting proper traffic control of the facility. Samples from the packaging line did not yield any *L. monocytogenes*.

### Facility P2 (105 Isolates: 1 Serogroup IIa, 96 of IVb-v1 and 8 of LIII)

In this facility, many samples yielded ST554/CC554 (IVb-v1) isolates that were likely the same strain; many samples yielded ST1003 (LIII) isolates that were likely the same strain.

#### Two Samplings in Fall 2016, September (F1) and October (F2)

(1) In the F1 sampling, 8 positive samples yielded 12 isolates.
  Two samples in the cold storage area were positive. Sample #2 from the floor 2/3 in the short-term storage and staging sub-area yielded 1 isolate (ST1507/CC1320, LIII); and sample #8 from the floor 2/3 in the long-term storage sub-area yielded 1 isolate (ST554/CC554).
  Six samples in the packing line were positive. Sample #22 from the drip-pan of the fan-dry sub-area yielded 1 isolate (ST554/CC554); sample #23 from the scraper bar and drip pan funnel below the fan-dry sub-area yielded 1 isolate (ST554/CC554); sample #24 from the floor below the fan-dry sub-area yielded 2 isolates (ST554/CC554 and ST1003); sample #27 from the drip pan of wax dripping site yielded 2 isolates (ST554/CC554 and ST1003); sample #28 from the structural support of wax dripping site yielded 3 isolates (ST554/CC554, ST1003 and ST1510, LIII); and sample #29 from the floor below waxing yielded 1 isolate (ST554/CC554).
(2) In the F2 sampling, 17 positive samples yielded 18 isolates.
  Sixteen samples in the packing line were positive. Among them, sample #30 from the catwalks adjacent to the packing line 1/2 (a potential cross-contamination site) yielded 1 isolate (ST1003); sample #27 from a drip pan/wax drip site yielded 2 isolates (ST554/CC554 and ST1003); and 14 samples (#17, #20, #21, #22, #23, #24, #25, #26, #28, #29, #31, #32, #33, and #34) from various sample sites of spray-wash, fan-dry, waxing, and potential cross-contamination sites each yielded 1 isolate, all of which were ST554/CC554.
  One sample (#38) from the sorting line equipment in the packaging line was positive and yielded 1 isolate (ST554/CC554).
(3) In summary, the ST554/CC554 strain apparently spread to multiple sub-areas in the cold storage, packing line, and packaging line areas of the facility, and persisted through the two successive samplings that fall. The ST1003 strain spread to multiple sub-areas in the packing line and persisted through the two consecutive fall samplings.

#### Two Samplings in Winter 2017, January (W1) and February (W2)

(1) In the W1 sampling, 24 positive samples yielded 25 isolates.
  Five samples in the cold storage area were positive. Sample #10 from a floor crack/seam in the long-term storage sub-area yielded 1 isolate (ST554/CC554). Others were in short-term storage and staging sub-area. Sample #1 from the floor 1/3 yielded 1 isolate (ST554/CC554); sample #2 from the floor 2/3 yielded 1 isolate (ST554/CC554); sample #3 from the floor 3/3 yielded 2 isolates (ST554/CC554 and ST37/CC37, IIa); and sample #5 from the floor crack/seam 2/2 yielded 1 isolate (ST554/CC554).
  Sixteen samples in the packing line were positive, yielding 16 isolates, all of which were ST554/CC554. These samples were #16, #20, #21, #22, #23, #24, #25, #26, #27, #28, #29, #30, #31, #32, #33, and #34, from various sample sites in the dump tank, spray-wash, fan-dry, waxing and potential cross-contamination sites (Table 1).
  Three samples from the packaging line were positive. Sample #37 from the floor below the sorting line yielded 1 isolate (ST554/CC554); sample #38 from a sorting line equipment yielded 1 isolate (ST554/CC554); and sample #39 from the floor below the packing line yielded 1 isolate (ST1003).
(2) In the W2 sampling, 24 positive samples yielded 24 isolates, all of which were ST554/CC554.
  Six samples (#2, #5, #7, #8, #9, and #10) were from various sample sites in the long-term and short-term storage sub-areas.
  Sixteen samples (#14, #20, #21, #22, #23, #24, #25, #26, #27, #28, #29, #30, #31, #32, #33, and #36) were from various sample sites of the dump tank, spray-wash, fan-dry, waxing and potential cross-contamination sites (Table 1).
  Two samples (#39 and #40) were from the line equipment and the floor below the packaging line.
(3) In summary, the ST554/CC554 strain was widespread among multiple sub-areas in the cold storage, packing line, and packaging line of the facility, and persisted through the two consecutive winter samplings.

#### Two Samplings in Spring 2017, May (S1) and July (S2)

(1) In the S1 sampling, 14 positive samples yielded 14 isolates, all of which were ST554/CC554.
  The three positive samples (#3, #7, and #10) were from various sample sites in the long-term and short-term storage sub-areas.
  The eleven positive samples (#20, #21, #23, #24, #27, #28, #29, #30, #31, #32, and #33) were from various sample sites of the spray-wash, fan-dry, wax, and potential cross-contamination sites in the packing line (Table 1).
(2) In the S2 sampling, 12 positive samples yielded 12 isolates, all of which were ST554/CC554.
  Eleven samples (#18, #20, #22, #23, #24, #26, #27, #28, #29, #30, and #32) were from various sample sites of the spray-wash, fan-dry, wax, and potential cross-contamination sites in the packing line.
  One sample (#38) was from the sorting line equipment in the packaging line.
(3) In summary, the CC554 strain spread widely to multiple sub-areas in the cold storage, packing line, and packaging line of the facility, and persisted through the two consecutive spring samplings.

#### Summary of P2

Overall, 105 isolates from 5 clones were collected in P2, resulting in a Shannon’s index of 0.38 (Figure 2). The CC554 strain spread widely to multiple sub-areas in cold storage, packing line, and packaging line of the facility, and persisted through all six samplings. The persistence of isolates in successive samplings suggested relatively poor sanitation conditions in that facility and is further discussed below.

### Facility P3 (18 Isolates: 6 Serogroup IIa, 2 IIb, 1 IVb, 1 IVb-v1, and 8 LIII)

#### Two Samplings in Fall 2016, September (F1) and November (F2)

(1) The F1 sampling did not yield any *L. monocytogenes*.
(2) In the F2 sampling, 3 samples, all in the packing line, yielded 3 isolates. Sample #23 from the scraper bars and the drip pan funnel below the fan-dry sub-area yielded 1 isolate (ST219/CC4, IVb), sample #27 from the drip pan/wax drip site yielded 1 isolate (ST1510, LIII) and sample #28 from the structural support/wax drip site below the waxing sub-area yielded 1 isolate (ST1510, LIII). Among these isolates, the ST1510 isolates from samples #27 and #28 were likely the same strain. Thus, different sampling sites in the waxing sub-area were likely contaminated by the same strain.

#### Two Samplings in Winter 2017, January (W1) and March (W2)

(1) In the W1 sampling, 8 samples yielded 12 isolates.
  Two samples from the cold storage area were positive. Sample #9 from the floor 3/3 in the long-term storage sub-area yielded 1 isolate (ST1508/CC20, IIa) and sample #4 from the floor crack/seam 1/2 in the short-term storage and staging sub-area yielded 1 isolate (ST5/CC5, IIb).
  Six samples from the packing line were positive. Sample #25 from the structural support/wax spray bar over waxing brushes yielded 1 isolate (ST1513, LIII); sample #27 from the drip pan/wax drip site yielded 2 isolates (ST1514, IIa, and ST1510, LIII); sample #28 from the structural support/wax drip site below the waxing line yielded 2 isolates (ST1513, LIII and ST1510, LIII); sample #29 from the floor below the waxing line yielded 1 isolate (ST1510, LIII); sample #31 from the catwalks adjacent to lines 2/2, a potential cross-contamination site, yielded 1 isolate (ST374/CC369, IIa); and sample #35 from a forklift tine, a potential cross-contamination site, yielded 3 isolates (ST1508/CC20, IIa; ST374/CC369, IIa and ST554/CC554, IVb-v1). Among these isolates, both ST1513 isolates from samples #25 and #28 were likely the same strain; the ST1510 isolates from samples #27, #28, and #29 were likely the same strain. Thus, multiple strains spread to different sample sites of the waxing sub-area.
  Both ST1508 isolates from samples #9 and #35 were likely the same strain, indicating transmission between the long-term storage sub-area and the packing line.
(2) The W2 sampling did not yield any *L. monocytogenes*.

#### Two Samplings in Spring 2017, May (S1) and July (S2)

(1) The S1 sampling did not yield any *L. monocytogenes*.
(2) In the S2 sampling, 3 positive samples yielded 3 isolates.

One sample (#5) from the floor crack/seam 2/2 in the short-term storage and the staging sub-area yielded 1 isolate (ST489, IIb).

Two samples in the packing line were positive. Sample #14 from a bin loading equipment near the dump tank yielded 1 isolate (ST1516/CC443, LIII) and sample #31 from the catwalks adjacent to packing line 2/2, a potential cross-contamination site, yielded 1 isolate (ST374/CC369, IIa).

#### Summary of P3

Overall, 18 isolates from 10 clones were collected in P3, resulting in a Shannon’s index of 2.10 (Figure 2). The ST1510 isolates collected in the F2 sampling and those collected in the W1 sampling likely belonged to the same strain, indicating persistence across two seasons. The CC369 isolates collected in the W1 sampling and those collected in the S2 sampling likely belonged to the same strain, indicating persistence across two seasons. Therefore, there were both transient contamination and short-term persistence in P3.

### Transient and Persistent Contamination and Inter-Area Transmission

In order to study the transmission events, we divided each facility to 3 areas, the cold storage, the packing line, and the packaging line (Table 1). WGS data indicated no cross-area transmission or cross-season persistence events in P1, and only short-term persistence between two samplings in the same season. However, within each area, cross-contamination in different sub-areas was detected. In the cold storage area, we sampled sites such as floors, floor cracks and the foot of fruit storage bins; cross-contamination among these sites, possibly caused by employee or equipment traffic, was detected during multiple samplings. In the packing line, spray-wash, fan-dry and/or waxing sub-areas were contaminated by the same strain(s) during multiple samplings, and in one sampling, such cross-contamination was likely due to the same strain present in the catwalks along the packing line. The drain where rinse water and product debris from spray-wash, fan-dry and waxing sub-areas were collected was contaminated with the same strain. Even though facility P3 had the lowest incidence of *L. monocytogenes* among the 3 facilities, our data indicated a cross-area transmission event and two cross-season contamination events, which were not observed in facility P1. We only sampled Zone 2 and Zone 3, and thus, we could not determine if there was cross-area transmission in Zone 1. Nonetheless, it appears that sanitation practices in P1 and P3 effectively eliminated most of *L. monocytogenes*, however, constant reintroduction of different strains occurred. In contrast, there were persistence and wide spread of *L. monocytogenes* in P2. This difference could be explained by relatively poor sanitation controls in P2 compared to P1 and P3 (Tan et al., 2019). As a result, Shannon’s indexes of clone diversity for P1 and P3 was 2.49 and 2.10, respectively and those were significantly higher (*p* < 0.05) than that of P2 (Shannon’s index, 0.38). In our other study that analyzed the relationship between the prevalence of *L. monocytogenes* and the microbiome of the 3 facilities during the following year (i.e., Year 2), P2 had the highest quantity of *L. monocytogenes* (i.e., 1.5–3 log higher than those in P1 and P3) and lowest bacterial and fungal species richness (Tan et al., 2019). The authors hypothesized that lower diversity of microbial species could lead to less competition for nutrients and less inhibition from competitor flora, which might render P2 an environment that supports the persistence of *L. monocytogenes* (Tan et al., 2019). Furthermore, biofilm formers and indicators of unsanitary conditions, such as Pseudomonadaceae and Dipodascaceae, were predominant in P2, and the authors hypothesized that *Pseudomonas* might have formed biofilms and sheltered *L. monocytogenes* from cleaning and sanitizing treatments (Tan et al., 2019). Indeed, our targeted sequencing of isolates collected in the hotspots of P2 at the end of Year 2 confirmed that the CC554 strain was still present in P2 (our unpublished data). No quantification and microbiome analysis were conducted in Year 1 when isolates discussed in this study were collected; however, it is not unreasonable to speculate that the quantity of *L. monocytogenes* and species richness in these 3 facilities were not significantly different between Year 1 and Year 2. In addition, since our enrichment-based detection could introduce bias toward finding the more abundant strain(s) in the environment, we cannot exclude the possibility that higher than what we observed diversity of *L. monocytogenes* was present in P2.

The ST1510 isolate from the P1 packing line collected during the W1 sampling likely belonged to the same strain as the ST1510 isolates from the P3 packing line collected during the F2 and W1 sampling. The CC37 isolates from the P1 short-term storage sub-area during the W1 sampling belonged to the same strain as the CC37 isolates from the P2 short-term storage sub-area in the W1 sampling. Therefore, common sources of contamination of these facilities could exist.

The wide spread of a *L. monocytogenes* strain in a facility was also observed in several recent listeriosis outbreaks, in which WGS performed during traceback investigations revealed that multiple locations of the facility were contaminated by the outbreak strain (Chen et al., 2016a,2017a,2017b,2017c).

### Distribution of Lineages, Serotypes, and Clones of *L. monocytogenes* in Facilities

**FIGURE 3:**
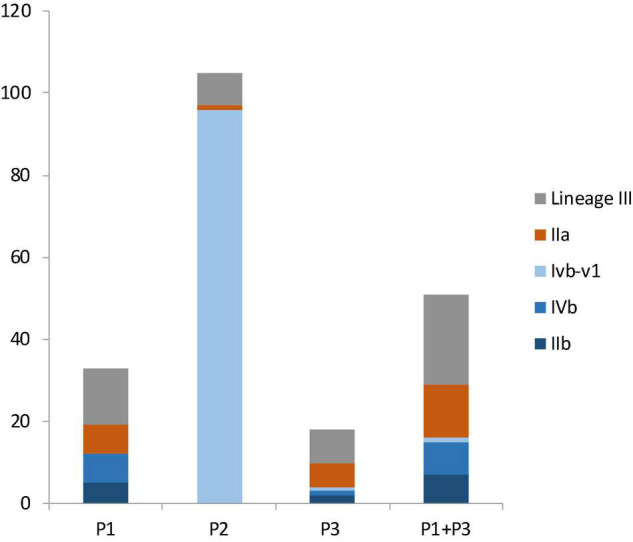
The number of isolates from different molecular serogroups in facility P1, P2, P3 and in both P1 and P3. Lineage III isolates, in gray color, were not assigned a serogroup. Serogroup IIa, in brown color, belongs to lineage II. Serogroups IIb, IVb, and IVb-v1, in three shades of blue colors, belong to lineage I.

Facility P1 had 12 lineage I isolates (incidence: 36.3%), 7 lineage II isolates (21.2%) and 14 lineage III isolates (42.4%); the difference was not significant (*p* > 0.05) (Figure 3). Facility P2 had 96 lineage I isolates (91.4%), all of which belonged to CC554, 1 lineage II isolate (1%) and 8 lineage III isolates (7.6%), 6 of which were ST1003; The wide spread of a single CC554 strain and modest spread of an ST1003 strain across facility P2 contributed to much higher incidence of lineage I and much lower incidence of lineage II and III in this facility (*p* < 0.05). Facility P3 had 4 lineage I isolates (22.2%), 6 lineage II isolates (33.3%) and 8 lineage III isolates (44.4%); the difference was not significant (*p* > 0.05) (Figure 2). When combining data from only facilities P1 and P3, lineage III isolates had the highest incidence (43.1%), followed by lineage I (31.3%) and lineage II (25.5%) isolates, even though the incidence was not significantly different (*p* > 0.05) (Figure 3). In previous studies, lineage III isolates were predominantly isolated from animal sources, and rarely isolated from food processing environments (Orsi et al., 2011). Thus, our findings of high prevalence of lineage III isolates in P1 and P3 may provide new insights on *L. monocytogenes* contamination in tree fruit packing environments. Perhaps there were connections between the contamination sources of these packinghouses and wild animals since lineage III of *L. monocytogenes* was often found in animals (Orsi et al., 2011).

Among lineage I isolates in facility P1 were 5 serogroup IIb isolates and 7 serogroup IVb isolates; among lineage I isolates in facility P3 were 2 serogroup IIb isolates, 1 serogroup IVb isolate, and 1 serogroup IVb-v1 isolate (Figure 3). When combing data of lineage I isolates from P1 and P3, there were 7 serogroup IIb isolates (43.8%), 8 serogroup IVb isolates (50%) and 1 serogroup IVb-v1 isolate (6.3%) (Figure 3); there was no significant difference between the occurrence of serogroup IVb and serogroup IIb (*p* > 0.05). All lineage II isolates in these facilities were serogroup IIa (Figure 3).

When combining data from P1 and P3, we did not observe any strong association between *L. monocytogenes* genetic lineages and facility areas (i.e., cold storage and packing line). No *L. monocytogenes* was isolated from packaging lines from P1 and P3.

In this study, *in silico* analysis of 7-gene MLST defined a total of 24 clones. Among them, 8 were novel clones that are first reported in this study, ST1507/CC1320, singletons ST1509, ST1510, ST1511, ST1512, ST1513, ST1514, and ST1515. In addition, two novel STs of existing clones were discovered, ST1516 of CC433 and ST1508 of CC20. Due to the varying degree of WGS diversity of a few MLST-defined clones, cgMLST diversity was proposed to redefine *L. monocytogenes*, and a new term, sublineage, was proposed (Moura et al., 2016). With the 1827-cgMLST scheme employed in the current study, isolates belonging to a typical MLST-defined clone differed by ≤ 167 alleles, and isolates belonging to a lineage I or II differed by ≤ ∼1,500 alleles (Chen et al., 2016b,2017c). In this study, isolates in all other clones except singleton ST1510 differed by ≤ 115 cgMLST alleles within each clone, thus, isolates in these clones would have been classified into the same clone if WGS diversity were used to define *L. monocytogenes* clones. In singleton ST1510, CFSAN056290 differed from the other 12 isolates by up to 540 alleles, and the other isolates differed by ≤ 29 alleles; thus, CFSAN056290 could belong to a different clone from the other 12 isolates if WGS diversity were used to define *L. monocytogenes* clones. Due to the relatively small number of isolates for each clone, we could not determine any strong association between clone and facility, except that CC554 dominated facility P2. The ST1512 isolates harbored ORF2819 in the molecular serogrouping scheme which indicated it was serogroup IIb, however, this singleton was determined to be lineage III according to the WGS phylogeny, therefore, we considered this multiplex PCR serogroup identification incorrect.

The clones associated with the 2014–2015 multistate listeriosis outbreak linked to caramel apples (i.e., CC1 and ST382) were not found in this study (Chen et al., 2017c). Among the clones identified in this study, CC4 was a hypervirulent clone previously associated with multiple listeriosis outbreaks outside United States (Chen et al., 2016b; Cabal et al., 2019); CC5 had been associated with multiple outbreaks associated with contaminated cheese, ice cream and cantaloupe (Chen et al., 2016b); CC554 had been associated with a 2014 multistate outbreak linked to contaminated mung bean sprouts (Centers for Disease Control and Prevention, 2015; Yang et al., 2020). The CC4, CC5 and CC554 isolates collected in this study did not match the strains of aforementioned outbreaks (our unpublished data). Several major hypervirulent clones, such as CC1, CC2, and CC6 (Maury et al., 2016), were not found in this study. This is consistent with observation in France that hypervirulent clones were not prevalent in foods and food processing environments.

Among isolates that were persistent in facility P2, we did not observe any significant temporal or spatial microevolution events of the CC554 isolates; isolates from different areas (i.e., cold storage, packing line and packaging line) did not form any specific clades, and isolates from different samplings did not form any specific clades. For most samples, multiple isolates from the same sample did not form a clade. In addition to the cgMLST targeting 1,827 genes, we also performed an analysis targeting the entire genome and reached the same conclusion (data not shown).

### Multiple Cases of Multi-Strain Contamination in the Same Sponge Sample Illustrate Challenges in *L. monocytogenes* Isolation Methods From Food Processing Environments and the Need for Analyzing Multiple Isolates per Sample

In this study, we used the 48 h enrichment cultures to analyze the environmental samples and picked up to 4 typical colonies from agar plates of each sample for identification and WGS. Fourteen out of 139 (10%) positive samples yielded more than one strain per sample. Different strains in each sample always belonged to different clones. Given the very large clonal diversity observed in P1 and P3, we speculate that more samples could be contaminated with more than one strain, and more clones might be present in these facilities, but the 48 h enrichment protocol we employed might confer disadvantage to strains that had less abundance. Perhaps, using shorter enrichment duration (e.g., 24 h) could lead to the detection of those less abundant strains, however, heavily stressed cells may not recover with shorter or no enrichment (Sheth et al., 2018). In addition, sampling more than 4 colonies per sample could also lead to the discovery of more strains. In our study, sampling of 40 locations per facility and picking of up to 4 isolates per sample could compensate the bias introduced by the 48 h enrichment, at least partially, since we expect that not every single location was co-contaminated by strains with differing abundance. Previous studies analyzing WGS data of isolates recovered using enrichment-based methods during outbreak traceback investigations also reported differing abundance of strains contaminating the food processing environments or foods. For example, the facility implicated in the 2014 stone fruit outbreak yielded a singleton ST382 strain from 3 environmental samples, a CC5 strain from 13 environmental samples and a singleton ST392 strain from only one sample, suggesting differing prevalence of these clones contaminating the facility (Chen et al., 2016a; Yang et al., 2020). Therefore, if the purpose of a study is to comprehensively survey all genotypes of *L. monocytogenes* in a food production environment, the detection methods need to be properly evaluated and sufficient number of locations need to be sampled. For another example, the CC5 outbreak strain was present predominantly in ice cream produced in a facility, but another non-outbreak CC5 strain was also isolated from a few ice cream samples produced in the same facility (Chen et al., 2017b). In that case, a simple 7-gene MLST-identified clone could not provide the precise picture of the genetic diversity of *L. monocytogenes* in that facility; rather, WGS was needed to provide the high resolution on *L. monocytogenes* genotypes.

### Presence of Premature Stop Codons of *inlA*, the Genes Associated With Virulence and Stress Resistance, and Plasmid

The presence of major virulence genes and genes implicated in stress response and environmental persistence (Moura et al., 2016; Maury et al., 2019; Chen et al., 2020) were determined for all the isolates (Figure 1). Only the CC5 isolate, CFSAN062927, contained a premature stop codon (PMSC) in *inlA*. PMSC in *inlA* has been associated reduced virulence of some *L. monocytogenes* strains (Nightingale et al., 2008). A 65 amino acid (AA) region (606th AA to 671th AA), part of the B-repeat (Ebbes et al., 2011), of the ST1514 isolate (CFSAN062934) was not covered by shotgun sequencing, possibly due to sequencing artifacts. However, the examination of the sequencing-covered *inlA* region of this isolate did not reveal any premature stop codons. Previous studies showed that *inlA* PMSCs occurred more frequently in lineage II isolates than in lineage I isolates and that isolates with *inlA* PMSCs were often overrepresented in food and environmental samples, but underrepresented in clinical samples (Van Stelten et al., 2010, 2016). In the present study, only one isolate contained a PMSC. This is similar to studies performed by Gorski et al. (2016) who reported that only 2.7% of *L. monocytogenes* isolates from naturally contaminated watersheds contained *inlA* PMSCs, Wang et al. (2015) who reported 2.4% of *L. monocytogenes* isolates from retail deli environments containing *inlA* PMSCs, and (Kim et al., 2018) who reported 2.5% of isolates from milk, milk filters and milking equipment from United States dairies containing *inlA* PMSCs. Therefore, analyses of additional strain collections should be performed to determine the overall prevalence of *inlA* PMSCs in food, environmental and clinical isolates.

*Listeria* pathogenicity island-1 (LIPI-1), the first identified *L. monocytogenes* genomic island where virulence factors cluster together (Vazquez-Boland et al., 2001), was found in all isolates. LIPI-3, encoding listeriolysin S, was found in CC4, CC288, ST489 and CC379 isolates. LIPI-4, a newly identified pathogenicity island associated with neural and placental infections (Maury et al., 2016), was found in CC4 and CC217. Major internalins (Moura et al., 2016; Chen et al., 2020), which were surface proteins that are associated with attachment of *L. monocytogenes* host cells (Navarre and Schneewind, 1999), were found in the isolates except for 3 internalin genes. Specifically, *inlC* and *inlF* were not found in a portion of lineage III isolates and *inlG* was not found in lineage I isolates. Many genes involved in resistance to low pH, high salt concentrations, desiccation, alkaline stress, and oxidative stress were found in all the isolates. *arcABC* were not found in any lineage III isolates, while *arcA* and *arcR* were found in all isolates. *arcABCDR* was involved in resistance to low pH (Matereke and Okoh, 2020). Several plasmid-borne benzalkonium chloride (BC) tolerance genes have been confirmed to contribute to adaptation of *L. monocytogenes* to food processing environments (Moura et al., 2016; Maury et al., 2019). Among them, *qacA*, *qacC*, *qacH*, *emrC*, and *emrE* were not found in any isolates; the *bcrABC* cassette was found in the only CC5 isolate. Therefore, these BC tolerance determinants were not associated with the occurrence of *L. monocytogenes* in the 3 tree fruit packinghouses. *Listeria* stress survival islet 1 (SSI-1), which was involved in growth of *Listeria* under suboptimal conditions such as low pH and high salt concentrations (Ryan et al., 2010), was found in 3 lineage I isolates (18.8% when only counting P1 and P3), 9 lineage II isolates (64.3% of all lineage II isolates), and 25 lineage III isolates (83.3% of all lineage III isolates). *Listeria* stress survival islet 2 (SSI-2), associated with *Listeria* alkaline and oxidative stress responses (Harter et al., 2017), which may contribute to the wide spread of CC121 in food production environments in several countries of Europe (Schmitz-Esser et al., 2015; Rychli et al., 2017; Pasquali et al., 2018; Maury et al., 2019), was found only in isolates of ST1513 and ST1512 which both belonged to lineage III. *Listeria* genomic island 2 (LGI2), which was associated with arsenic and cadmium resistance and previously found in several hypervirulent clones of serotype 4b (Lee et al., 2017), was found only in isolates of CC331, lineage III. Thus, quite a few lineage III isolates contained genes associated with *Listeria* stress resistance. Another cadmium resistance cassette, *cadA2C2*, was found only in the CC5 isolate. Two genes involved in biofilm formations, *inlL* and *bapL*, were found only in isolates of lineage II. The only CC5 isolate, CFSAN062927 contained a plasmid which was nearly identical to the LM-F-131 plasmid (NCBI Accession, CM009923.1, 81.7Kbp), containing *bcrABC* and *cadA2C2*. Five other isolates contained plasmid contigs that partially matched the plasmid pLMIV (NCBI Accession, CM001470.1, 77.8 Kbp) of a lineage IV isolate (J1-208) and did not match any other plasmids deposited in GenBank as of November 10th, 2019. Specifically, the 3 CC369 (lineage II) isolates each contained plasmid contigs of ∼48 Kbp; the CC20 (Lineage II) isolate, CFSAN058395, contained plasmid contigs of ∼34 Kbp; and a ST1510 isolate, CFSAN056290, contained plasmid contigs of ∼61 Kbp. Plasmid pLMIV did not contain known heavy metal resistance genes or benzalkonium chloride resistance genes. This plasmid analysis corroborates our results showing that only CFSAN062927 contained known plasmid-borne stress resistance genes.

The widely spread CC554 strain in P2, which persisted for at least 2 years based on data in the present study and our unpublished data, did not contain the SSI-1, SSI-2, LGI2, any other heavy metal resistance genes, or any genes associated with BC tolerance.

The genes associated with biofilm formation found in the CC554 strain were also found in all other isolates. This indicated that the persistence of this CC554 strain might not be strongly associated with these identified stress resistance mechanisms; other factors, such as poor sanitary conditions, the protection from biofilm-forming background flora, and/or unidentified stress resistance mechanisms might have played major roles in the persistence of CC554.

### Growth in the Presence of Low Concentrations of Sanitizer

The strains chosen to measure the resistance to Sani-T-10 were CFSAN062890 (ST1003), CFSAN062899 (ST1511), CFSAN062927 (CC5), CFSAN062933 (ST1513), CFSAN062942 (ST1510), CFSAN062946 (CC20), CFSAN062947 (CC369), and CFSAN071674 (CC554), and their MICs were 2, 2, 3, 3, 3, 2, 3, and 3 ppm, respectively. These strains were chosen to represent lineages I, II and III; and those strains that were repeatedly isolated in facility P2 (i.e., CFSAN062890 and CFSAN071674). In previous studies, many *L. monocytogenes* strains that contained BC tolerance genes and those that were persistent in food processing environments exhibited 2–4-fold increased resistance to low levels of BC-based sanitizers (Carpentier and Cerf, 2011; Muller et al., 2014; Ortiz et al., 2016; Moretro et al., 2017; Harrand et al., 2020). In our study, only one strain (CFSAN062927) contained BC tolerance genes and only two strains (CFSAN062890 and CFSAN071674) might have been persistent in the packing environment; and these three strains did not show increased resistance to low concentrations of Sani-T-10 compared to other isolates. Therefore, sanitizer resistance might not be a factor that contributed to the presence of these three strains in the packinghouses.

## Conclusion

High resolution of WGS analysis of *L. monocytogenes* from 3 apple packinghouses collected in 1 year revealed the clonal diversity of *L. monocytogenes* and allowed the detection of transient contamination, persistent contamination, and cross-area transmission events. Different facilities exhibited distinct contamination patterns (i.e., persistent vs. transient, widespread vs. localized). The facility P2, with the poorest sanitary conditions, had the least diversity of *L. monocytogenes* clones; P2 contained a CC554, serogroup IVb-v1 strain persisting through the year and spreading across entire facility, an ST1003, lineage III strain persisting through two seasons and spreading across two areas of the facility, and several other strains due to transient contaminations. The facilities with better sanitary conditions, P1 and P3, had much higher diversity of *L. monocytogenes* clones, as a result of transient contamination, with little cross-area spread in the facilities.

The collected isolates exhibited unique genotypic characteristics. In facilities P1 and P3, lineage III isolates, which were predominantly isolated from animal sources previously, had the highest incidence (43.1%), followed by lineage I (31.3%) and lineage II (25.5%), indicating possible connection between animal sources and contamination sources of these two facilities. Only one isolate contained premature stop codons in *inlA*. The persistent strain CC554 did not contain major genes that are confirmed molecular determinants of *L. monocytogenes* stress resistance, and did not exhibit stronger sanitizer resistance than other isolates, demonstrating that persistence of *L. monocytogenes* in a packinghouse could be determined by multiple factors.

## Data Availability Statement

The datasets presented in this study can be found in online repositories are deposited in NCBI. The names of the repository/repositories and accession number(s)/biosample IDs can be found in the article/Supplementary Material.

## Author Contributions

YC: methodology, data analysis, and writing of the manuscript. TS: data collection. KP and LL: methodology, review, and editing. QJ: laboratory analysis, data collection, and analysis. EB: supervision of data collection and analysis. DM: conception and administration of the project, methodology, review, and editing. All authors contributed to the article and approved the submitted version.

## Conflict of Interest

The authors declare that the research was conducted in the absence of any commercial or financial relationships that could be construed as a potential conflict of interest.

## Publisher’s Note

All claims expressed in this article are solely those of the authors and do not necessarily represent those of their affiliated organizations, or those of the publisher, the editors and the reviewers. Any product that may be evaluated in this article, or claim that may be made by its manufacturer, is not guaranteed or endorsed by the publisher.

## Footnotes

1
https://bigsdb.pasteur.fr/listeria/listeria.html
